# Comparison of five supervised feature selection algorithms leading to top features and gene signatures from multi-omics data in cancer

**DOI:** 10.1186/s12859-022-04678-y

**Published:** 2022-04-28

**Authors:** Tapas Bhadra, Saurav Mallik, Neaj Hasan, Zhongming Zhao

**Affiliations:** 1 Department of Computer Science and Engineering, Aliah University, Kolkata, West Bengal 700160 India; 2 Center for Precision Health, School of Biomedical Informatics, The University of Texas Health Science Center at Houston, Houston, TX 77030 USA; 3 Human Genetics Center, School of Public Health, The University of Texas Health Science Center at Houston, Houston, TX 77030 USA

**Keywords:** Feature selection, Multi-omics data, Classifier, Representation entropy, Redundancy rate

## Abstract

**Background:**

As many complex omics data have been generated during the last two decades, dimensionality reduction problem has been a challenging issue in better mining such data. The omics data typically consists of many features. Accordingly, many feature selection algorithms have been developed. The performance of those feature selection methods often varies by specific data, making the discovery and interpretation of results challenging.

**Methods and results:**

In this study, we performed a comprehensive comparative study of five widely used supervised feature selection methods (mRMR, INMIFS, DFS, SVM-RFE-CBR and VWMRmR) for multi-omics datasets. Specifically, we used five representative datasets: gene expression (Exp), exon expression (ExpExon), DNA methylation (hMethyl27), copy number variation (Gistic2), and pathway activity dataset (Paradigm IPLs) from a multi-omics study of acute myeloid leukemia (LAML) from The Cancer Genome Atlas (TCGA). The different feature subsets selected by the aforesaid five different feature selection algorithms are assessed using three evaluation criteria: (1) classification accuracy (Acc), (2) representation entropy (RE) and (3) redundancy rate (RR). Four different classifiers, viz., C4.5, NaiveBayes, KNN, and AdaBoost, were used to measure the classification accuary (Acc) for each selected feature subset. The VWMRmR algorithm obtains the best Acc for three datasets (ExpExon, hMethyl27 and Paradigm IPLs). The VWMRmR algorithm offers the best RR (obtained using normalized mutual information) for three datasets (Exp, Gistic2 and Paradigm IPLs), while it gives the best RR (obtained using Pearson correlation coefficient) for two datasets (Gistic2 and Paradigm IPLs). It also obtains the best RE for three datasets (Exp, Gistic2 and Paradigm IPLs). Overall, the VWMRmR algorithm yields best performance for all three evaluation criteria for majority of the datasets. In addition, we identified signature genes using supervised learning collected from the overlapped top feature set among five feature selection methods. We obtained a 7-gene signature (*ZMIZ1, ENG, FGFR1, PAWR, KRT17, MPO* and *LAT2*) for EXP, a 9-gene signature for ExpExon, a 7-gene signature for hMethyl27, one single-gene signature (*PIK*3*CG*) for Gistic2 and a 3-gene signature for Paradigm IPLs.

**Conclusion:**

We performed a comprehensive comparison of the performance evaluation of five well-known feature selection methods for mining features from various high-dimensional datasets. We identified signature genes using supervised learning for the specific omic data for the disease. The study will help incorporate higher order dependencies among features.

## **Background**

So far, pattern recognition techniques have huge impacts in solving most of the complicated real-life problems such as motif discovery from sequence features, detection of gene signatures for disease status, among others [1–4]. These methodologies are applied either separately or in conjunction with various soft-computing tools such as neural networks, evolutionary computing, swarm intelligence, etc. to solve various problems. Dealing with biological problems has revealed many challenges from real and complex data [5–7].

In general, a pattern recognition scheme involves three stages: data acquisition, dimensionality reduction, and classification or clustering [8–10]. In many pattern mining applications, the data acquisition stage yields huge number of features, some of which may be unimportant and redundant. Thus, the dimensionality of the data needs to be reduced by removing those unimportant and redundant features. The subset of selected features thus obtained keeps the optimal salient characteristics of the data as much as possible. In this way, dimensionality reduction accelerates the process of knowledge discovery to achieve better pattern recognition tasks such as classification and clustering [11–13], etc.

The dimensionality of data can generally be reduced using two methods: feature selection and feature extraction [8, 14]. The feature selection methodologies selects a small subset of features that are most relevant to the class variable. On the other hand, the feature extraction methodologies acquire a new subset of features by taking various functions on the original features. As the attribute selection methodologies retain the overall originality of attributes when decreasing the number of attributes, they are often preferred in several cases where the chosen attributes require to be interpreted by the domain experts [15]. There are many applications for feature selections such as disease status prediction, microRNA transcription start site (TSS) prediction [16, 17], etc. Analyzing micro-array cancer data is one of the most promising applications of feature selection.

Feature selection can be accomplished in three ways, viz., supervised, semi-supervised and unsupervised [18]. Supervised approaches evaluate each candidate feature or feature subset by knowing class labels, while the unsupervised approaches derive some alternative criteria without knowing any class labels [19, 20]. The methods of feature selection can also be classified into three categories: filter, wrapper, and embedded, depending upon how the feature evaluation indicators are computed [21]. Filter-based methods assess each candidate subset of features based on some internal data evaluation metrics. These approaches of filter-based feature selection can again be classified into two groups, namely, scalar feature selection and feature vector selection. In a scalar feature selection plan, individual features are assessed based on some statistical criteria and eventually some user-specific number of top-ranked features are chosen. The features chosen through these approaches are remarkably strong but are less robust because they do not consider the inter-dependencies between features while choosing the desired number of features. Furthermore, there is no previous information about the accurate number of features that are considered in leading to offer better classification accuracies.

**Fig. 1:**
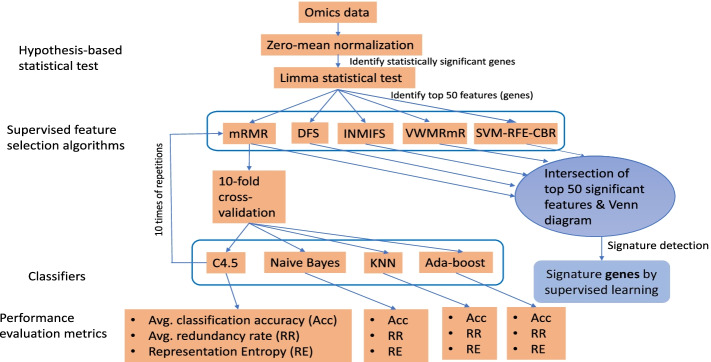
Pipeline of the proposed method

For the purpose of feature selection, collective knowledge is suggested over many alternative criteria such as Pearson correlation coefficient to compute the value (usefulness) of a feature subset due to its capacity of analyzing nonlinear dependency. Among all these measures, mutual information has appeared as one of the most potential criteria to solve the task of feature section. For example, Mutual Information based Feature Selection (MIFS) [22], Mutual Information Feature Selector under Uniform information distribution (MIFS-U) [23], minimal Redundancy Maximal Relevance criterion-based feature selection (mRMR) [24], Normalized Mutual Information- based Feature Selection (NMIFS) [25], Improved Normalized Information-based Feature Selection (INMIFS) [26], Variable Weighted Maximal Relevance minimal Redundancy criterion-based feature selection (VWMRmR) [27], are some well-known algorithms of the mutual information-based approaches. In this connection, a two-stage feature selection method is also used where a reasonably small number of features are first selected from a high dimensional data using the mRMR criterion, and then a better feature subset of features is selected from that candidate sets using a wrapper approach. In contrast to the filter-based approaches, the wrapper approaches employ the classification performance to choose the best subset of the reduced features. The value (usefulness) of feature subsets depends on selected types of classification models are used to measure the classification performances. Besides these, there are several other feature algorithms such as Support Vector Machine Recursive Feature Elimination (SVM-RFE) [28], Support Vector Machine Recursive Feature Elimination along incorporating the Correlation Bias Reduction (SVM-RFE-CBR) [29], Discriminative Feature Selection (DFS) [30], etc. However, the pipeline of this study is provided in Fig. 1.

As already mentioned, the feature extraction methods transform all the features of a given data into a new transformed feature space from which the originality of the features cannot be found. On the other hand, the feature selection methods select a small subset of features, where the originality of the selected features remains the same as it was before doing the feature selection. Therefore, the feature selection methods are most suitable for generating gene signatures by selecting a smaller subset of genes, while the feature extraction methods cannot obtain gene signatures.

In this research work, we considered five different state-of-the-art methods of feature selection for gene signature detection, such as mRMR, DFS, INMIFS, SVM-RFE-CBR, and VWMRmR. We assessed the their performance on various omics datasets in terms of several evaluation criteria. The experimental results showed that the average classification accuracy rate of the selected feature subset obtained using the VWMRmR method was superior when compared with other feature selection algorithms. The experimental results further showed that the subset of features selected using the VWMRmR method is prominent in terms of two other criteria, namely, average redundancy rate and representation entropy among all methods. Finally, we applied these five feature selection methods to a multi-omics dataset in acute myeloid leukemia, and performed the intersection operation among the sets of the selected top statistically significant genes (features) obtained by those methods to identify gene signatures from each omic data. Of note, MIFS (1994), MIFS-U (2002) and NMIFS (2009) were fundamental baseline feature selection algorithms that were very old. The mRMR (2005) method is an improved version of MIFS (1994) and MIFS-U (2002). On the other hand, another improved latest version of NMIFS called INMIFS (2010) was already developed. Thus, instead of considering the very oldest fundamental methods, we chose the improved version of them for our study.

## **Methods**

Currently, dimensionality reduction techniques are becoming more important due to the advancement of bigdata (i.e., high-dimensional datasets). Among various techniques, feature selection has become an emerging tool to identify the most suitable features in big data, both in terms of the number of samples or the number of features. Some of the widely used feature selection techniques are demonstrated below.

### **MIFS**

Battiti (1994) proposed the Mutual Information-Based Feature Selection (MIFS) criterion, which is defined as follows.

1 $$\begin{aligned} I (fi; C) -\beta \sum _{f_s\epsilon S}{I\ (f_s;f_i)}. \end{aligned}$$

The selection criterion is utilized to greedily pick the most notable *m* attributes from a collection of *d* attributes [22]. Provided a set of already selected features *S*, it is used to select a candidate feature $f_i$ which makes the most of the relevance to the class *I*(*fi*; *C*) at each stage without taking into account the joint mutual information between the selected attribute set and the class variable *C*. The parameter $\beta$ has a significant impact on the selected feature set. In the cases where $\beta$ is too big, it will enlarge the redundancy in the selected features. As a result, the MIFS method may remove redundant features that have high relevance with the class variable. The main disadvantage of the MIFS method is the selection of an ideal value for the parameter $\beta$.

### **MIFS-U**

Kwak and Choi developed an enhanced version of MIFS called as MIFS-U, which is based on the assumption that information is handled uniformly with specific features *S* [23]. The criterion which is used to select the promising feature at each stage is defined as follows.

2 $$\begin{aligned} I (f_i; C) -\beta \sum _{f_s\epsilon S}\frac{I\ \left( f_s;C\right) }{H\ (f_s)} I (f_i; f_s), \end{aligned}$$

where $H (f_s) = \sum _{f_s\epsilon s}{P\ (f_s)}\log {P\ (f_s)}$ is the entropy of $f_s$. The uniform probability distribution assumption can ensure conditioning by the class *C* does not alter the proportion of the mutual information between $f_s$ and $f_i$, and the entropy of $f_s$.

The MIFS-U method gauges a superior assessment of the MI measure than the MIFS method, however, it additionally needs to pick the parameter $\beta$ cautiously. With an inappropriate value of $\beta$, the MIFS-U method may provide poor results.

### **mRMR (minimum redundancy maximum relevance)**

Peng et al. proposed a parameter-free feature selection method called minimal Redundancy Maximum Relevance-based feature selection algorithm (mRMR) [24]. The mRMR method replaces the $\beta$ used in the selection criterion of the earlier methods MIFS and MIFS-U by $\frac{1}{|S|}$, where |*S*| is the size of the set *S* of already selected features. The selection criterion of the mRMR method is defined as follows.

3 $$\begin{aligned} I(f_i; C) -\frac{1}{|S|}\sum _{f_s\epsilon S} {I (f_i; f_s)}. \end{aligned}$$

The main advantage of the mRMR method over the MIFS, MIFS-U methods is that it does not require any parameter like $\beta$ used in their methods.

Feature selection, one of the fundamental issues in pattern recognition as well as machine learning, detects subgroups of data which are important to the parameters used here and is generally denoted as Maximum Relevance. These subsets involve the material that is pertinent but redundant and mRMR attempts to fix its target on this issue through providing those redundant subsets. mRMR has a different variation of forms in many parts alike speech recognition as well as cancer diagnosis. Features can be selected in different ways. One strategy is to select features which correlate strongest to the transportation variable. This has been termed as peak-purpose selection. Many heuristic approaches can be used, such as the sequential backward, forward, or floating selections. On the other scenario, features can be detected to be mutually that is far away from each other when still possessing parallel to the class-variable. This strategy, denoted as Minimum Redundancy Maximum Relevance (mRMR) has been found to be more sophisticated and efficient than the maximum relevance selection. In a specified case, the “correlation” can be updated by the statistical dependency between the variables. Mutual information can be used to estimate the dependency. In that scenario, it is highlighted that mRMR is basically an approximation to maximize the dependency between the joint handling of the chosen features and the distribution variable.

### **NMIFS (normalized mutual information feature selection)**

Estevez et al. enhanced a new version of mRMR, termed as Normalized Mutual Information Feature Selection method (NMIFS). The NMIFS method used the normalized mutual information to compute the redundancy between the selected features. The selection criterion of the NMIFS method is defined as follows [25]:

4 $$\begin{aligned} I(f_i; C) -\frac{1}{|S|}\sum _{f_s\epsilon S}{\hat{I}} (f_i; f_s), \end{aligned}$$

where ${\hat{I}}$, the normalized mutual information, is demonstrated as

5 $$\begin{aligned} {\hat{I}} (f_i; f_s) =\ \frac{I\ (f_i;f_s)}{\min {(H\left( f_s\right) ,\ H\ \left( f_i\right) )}}. \end{aligned}$$

Using the normalized mutual information measure when computing the feature-feature redundancy, the NMIFS method seeks to overcome the problem of imbalance between the relevance and redundancy terms of the selection criteria used in the MIFS, MIFS-U and mRMR techniques. However, the NMIFS method has some drawbacks. For binary-class problems, the entropy of the class variable is one if the distributions of the class variable are the same. But, if the difference between the two terms is large, the entropy value will be less than one, resulting in a discrepancy between these two terms of the selection criterion. For multi-class problems, the value of the left term is usually more than one, while the value of the right hand term is below one. The imbalance between these two terms may sometime force the feature selection algorithm to select a feature based on the maximal value of the left-hand term while neglecting the right-hand term. Thus, the NMIFS method may often fail to properly select the desired set of features due to the imbalance problem.

### **INMIFS (improved normalized mutual information feature selection)**

Vinh et al. proposed an improved version of the NMIFS method called Improved Normalized Mutual Information Feature Selection (INMIFS) method [26]. They used normalized mutual information to compute both the class-relevance and feature-feature redundancy. The selection criterion of the INMIFS method is shown below.

6 $$\begin{aligned} {\hat{I}}(C; f_i) -\ \frac{1}{|S|}\sum _{f_s\in S}{{\hat{I}}(f_s;f_i)}, \end{aligned}$$

where ${\hat{I}}$ denotes the normalized mutual information.

The main advantage of the INMIFS method over the NMIFS method is that it attempts to make both the left and right-hand terms of the selection criterion comparable in magnitude. Vinh et al. (2010) obtained better results using the INMIFS method compared to the NMIFS method for six multi-class datasets.

### **Discriminative feature selection (DFS)**

Hong Tao et al. developed a filter-based feature selection technique through combining Linear Discriminant Analysis (LDA) and sparsity regularization [30]. To perform the feature selection, they utilized row sparsity on the LDA transformation matrix by $l_{2,1}$-norm regularisation, and the outcome formulation optimizes to choose the most discriminating features while simultaneously discarding the redundant ones.

They could use the $l_{2,1}$-norm centred formulation to the $l_{2,p}$-norm regularised scenarios, providing them more *p* options for fitting the wider range of sparsity necessity. They proposed an efficient algorithm to resolve the $l_{2,p}$-norm minimization problem and highlighted that their proposed technique is able to reduce the target in a monotonic manner prior to convergence while $0 < p \le 2$. In addition, their method maintains the power for selecting the most discriminative features while also discarding the redundant ones.

### **SVM-RFE-CBR (support vector machine recursive feature elimination with correlation bias reduction)**

Yan et al. proposed an improved version of SVM-RFE called as Support Vector Machine Recursive Feature Elimination with Correlation Bias Reduction (SVM-RFE-CBR) by incorporating the correlation bias reduction (CBR) strategy into the feature elimination process [29]. Like SVM-RFE, the SVM-RFE-CBR method adopts a backward elimination strategy. And so, it is able to model the dependencies between features. They showed that the SVM-RFE-CBR performed better than SVM-RFE and many other common methods. The main advantage of SVM-RFE-CBR over SVM-RFE is that the former can handle feature sets having highly correlated features, while the latter one cannot do it. They further suggested that the stability of SVM-RFE-CBR could be improved by applying some ensemble methods.

### **VWMRmR (variable weighted maximal relevance minimal redundancy)**

Bandyopadhyay et al. developed another normalized mutual information-based feature selection algorithm called Variable Weighted Maximal Relevance minimal Redundancy criterion based feature selection technique (VWMRmR) [27]. Here, features are selected depending upon the weighted maximum relevance minimum redundancy strategy by which the relative weight of the redundancy is linearly increased with concern to the number of already selected features. The selection criterion of this technique is shown below:

7 $$\begin{aligned} \acute{I}(C;f_i)-(1+\frac{w*|S|}{k})*\frac{1}{|S|}\sum _{f_s \in S}\acute{I}(f_i;f_s), \end{aligned}$$

where $\acute{I}(f_i;f_s)$ is the normalized mutual information between two features: $f_i$ and $f_s$ ($\in S$), *w* denotes a user-specified positive, real-valued parameter which controls the relative weight of redundancy relative to the relevance, *S* is the set of already chosen features, and *k* is the number of the features that needs to be selected.

## **Comparison of different feature selection algorithms on multi-omics data**

Here, we used five state-of-the-art feature selection algorithms, viz., mRMR, DFS, INMIFS, SVM-RFE-CBR and VWMRmR to select a small subset of relevant features corresponding to multi-omics datasets. For each dataset, the top-ranked 50 features are selected for each of the five feature selection algorithms.

### **Used datasets**

For analysis, five datasets, namely, gene expression (Exp), exon expression (ExpExon), copy number variation (Gistic2), DNA methylation (hMethyl27) and Pathway activity dataset (Paradigm IPLs) from a multi-omics dataset of acute myeloid leukemia (LAML) from The Cancer Genome Atlas (TCGA) were acquired from the publicly available repositories [31, 32]. Each of these five datasets has three types of class labels denoting three different stages of diseases, such as favorable (31 samples), intermediate/ normal (96 samples), and poor (34 samples). Each dataset consists of hundreds to thousands continuous features obtained through different biological experiments. There are a total of 161 common samples and among all these five data profiles.

### **Normalization and statistical test**

We first started working with common set of genes and samples among all these five profiles. Each profile consisting of the common gene set was normalized gene-wise through the zero-mean normalization [33, 34] that was formulated below:

8 $$\begin{aligned} x_{i}^{norm}=(x_{i}-\mu )/ \sigma , \end{aligned}$$

where $\mu$ refers to the mean of the scores of all samples corresponding to the specific gene before normalization and $\sigma$ denotes the standard deviation of the scores of all samples corresponding to that gene before normalization; while $x_{i}$ and $x_{i}^{norm}$ signify the scores of i-th sample of that corresponding gene prior to and after normalization, respectively.

We then performed statistical test using Limma within the R statistical package [33, 35], which employs Empirical Bayes test gene-wise on the respective normalized data. We obtained different sets of statistically significant genes having p value < 0.05 for each profile.

### **Discretization and computation of mutual information**

As all attribute variables contain continuous types of values, each of them was pre-processed by converting into a Z-score (signifying zero mean and units). Each attribute variable is then was then discretized into three states, such as − 1, 0, and 1. If the value of the attribute for a given sample is less than the standard deviation of the attribute, it takes − 1; if the value of the attribute is greater than the standard deviation, it takes 1; and it takes 0 in all other cases. In this way, all the attributes are transformed into a discretized space from a continuous space. Then the mutual information between two features is easily computed in the discretized space.

### **Supervised feature selection**

After discretization, we applied those five earlier mentioned supervised feature selection strategies (viz., mRMR, INMIFS, DFS, SVM-RFE-CBR and VWMRmR) on each of the five data profiles, individually, and extracted 50 top significant features among all the statistically significant genes (features).

### **Used classifiers**

For experimental purposes, we used four classifiers, viz., C4.5, Naïve-Bayes, KNN and AdaBoost to assess the quality of the reduced feature subsets selected (i.e., 50 top statistically significant features here) using different feature selection algorithms, and then estimated the different evaluation metrics.

### **Evaluation method**

Weka [36] software is employed to make all four classifiers (C4.5, Naïve-Bayes, KNN and AdaBoost). The third classifier KNN rule has a user-defined parameter K whose value is determined as the rounded value of the square root of the number of objects in the training data. On the contrary, Naive Bayes considers that the features are normally distributed whose means and variances are determined from the data. The worth of these classifiers are assessed by reporting the average classification accuracies obtained by first applying ten-fold cross validation to the training data, then by repeating the same process ten times and finally by averaging these ten results to obtain a single estimation.

### **Evaluation criteria**

For each of the above four classifiers, the performance of the corresponding classification model related to a omics datasets is assessed in terms of three evaluation criteria, viz., Accuracy, Redundancy Rate and Representation Entropy. The first measure is a supervised measure, while the remaining two are unsupervised measures.

#### **Accuracy**

The accuracy (Acc) is computed as follows:

9 $$\begin{aligned} Acc =\frac{(TP+TN)}{(TP+TN+FP+FN)}, \end{aligned}$$

where *TP*, *TN*, *FP* and *FN* stands for the number of true positives, the number of true negatives, the number of false positives and the number of false negatives, respectively.

#### **Redundancy rate**

For a feature set *F* having *d* number of features, the redundancy rate of *F* (*RR*) is defined as follows.

10 $$\begin{aligned} RR = \frac{2}{d*(d-1)} \sum _{f_i,f_j \in F, j > i} Sim(f_i, f_j), \end{aligned}$$

where $Sim(f_i,f_j)$ is the similarity between two features $f_i, f_j \in F$. To compute the similarity, normalized mutual information, correlation coefficient, etc. can be used as the underlying measures.

#### **Representation entropy**

For a feature set *F* with *d* number of features, assume, the eigenvalues of the $d*d$ covariance matrix of *F* be $\uplambda _j$, $j = 1 \ldots d$. The representation entropy of *F*, denoted by $R\_Entropy$, is defined as follows.

11 $$\begin{aligned} R\_Entropy = -\sum _{i=1}^{s} \widetilde{\uplambda _i} \log \widetilde{\uplambda _i}, \end{aligned}$$

where $\widetilde{\uplambda _j}, j=1,\ldots ,s,$ are calculated as follows.

12 $$\begin{aligned} \widetilde{\uplambda _i} = \frac{\uplambda _i}{\sum _{i=1}^{d}\uplambda _i}. \end{aligned}$$

The $R\_Entropy$ attains its minimum value (zero) when all the eigenvalues but one are equal to zero, that is, when it contains all its information in one coordinate direction. However, if all the eigenvalues are equal, then the information is evenly distributed among all the features; thus, the $R\_Entropy$ will reach its maximum value. Thus, we have uncertainty in feature reduction. This measure is known as the representation entropy.

## **Results and discussion**

### **Experimental results**

This subsection first describes the overall performance analysis. Subsequently, the comparative performance of five different feature selection algorithms are summarized. Then, the comparative performance of different feature selection algorithms are assessed in terms of redundancy rate and representation entropy.

#### **Differential analysis**

Fundamental descriptions (#common samples, #classes, #statistically significant features) of each of these five profiles were described in Table 1.

**Table 1:** Fundamental characteristics such as statistically significant features, number of samples and number of classes among samples for the used datasets

Data profile	# classes	# statistically significant features	# samples
Exp	3	728	161
ExpExon	3	1100	161
hMethyl27	3	272	161
Gistic2	3	904	161
Pathway activity data	3	265	161

#### **Comparison of different feature selection algorithms in terms of classification accuracy**

We have evaluated the performances of the five existing feature selection algorithms - mRMR, DFS, INMIFS, SVM-RFE-CBR and VWmRMR, on the above mentioned five datasets, viz., Exp, ExpExon, Gistic2, hMethyl27 and Pathway activity. The average scores at the 10-fold cross validation with 10 times repetitions obtained by applying different classifiers to the reduced feature subsets selected using the five different feature selection algorithms across LAML datasets are reported in Table 2.

**Table 2:** Comparison of classification accuracies (with 10-fold cross-validation and 10 times repetitions each case) determined by various feature subsets selected through various feature selection algorithms

Dataset	Classifier	Feature selection algorithm				
		mRMR	INMIFS	DFS	SVM-RFE-CBR	VWMRmR
		Avg. (std)	Avg. (std)	Avg. (std)	Avg. (std)	Avg. (std)
Exp	C4.5	72.67(3.29)	73.98(2.08)	72.73(2.08)	**74.34(3.35)**^a^	73.29(3.06)
	Naive Bayes	83.04(0.88)	83.66(0.83)	80.94(0.72)	**86.52(0.66)**^a^	84.47(0.83)
	KNN	**89.19(0.52)**^a^	88.01(0.66)	84.35(1.16)	86.15(0.51)	88.01(0.83)
	AdaBoost	81.86(1.30)	81.43(1.42)	74.47(2.14)	**85.16(1.19)**^a^	81.37(0.97)
ExpExon	C4.5	77.08(1.69)	75.09(2.72)	73.23(3.43)	75.03(2.76)	**77.45(1.73)**^a^
	Naive Bayes	**86.27(0.62)**	85.22(0.57)	84.10(0.43)	85.90(0.59)	85.46(0.60)
	KNN	87.58(0.65)	87.45(1.01)	87.01(1.19)	84.35(0.39)	**87.83(0.32)**^a^
	AdaBoost	84.03(1.17)	82.24(1.99)	77.20(1.44)	83.35(0.71)	**84.47(1.24)**^a^
hMethyl27	C4.5	72.05(2.27)	70.87(1.26)	70.99(1.94)	**72.17(2.55)**^a^	71.99(2.18)
	Naive Bayes	76.21(1.01)	78.82(1.26)	80.94(1.28)	80.68(1.39)	**80.99(1.69)**^a^
	KNN	82.48(1.09)	83.48(0.89)	82.80(0.83)	**84.41(0.99)**^a^	83.23(0.97)
	AdaBoost	74.29(1.74)	77.89(1.38)	77.52(2.41)	78.14(1.77)	**80.56(1.58)**^a^
Gistic2	C4.5	**73.60(1.53)**^a^	68.01(2.45)	71.49(1.42)	72.55(0.76)	67.95(2.07)
	Naive Bayes	**78.32(0.85)**^a^	76.89(1.05)	75.84(0.46)	78.07(0.42)	77.20(1.02)
	KNN	76.02(0.73)	75.22(0.85)	74.10(0.42)	**76.52(1.27)**^a^	74.84(0.60)
	AdaBoost	**78.70(0.72)**^a^	78.57(0.73)	75.84(0.80)	78.20(0.46)	77.33(0.98)
Pathway	C4.5	**70.50(1.83)**^a^	66.65(2.70)	68.82(3.28)	64.97(2.72)	66.89(3.26)
activity	Naive Bayes	79.44(1.19)	82.30(1.56)	66.65(0.78)	76.83(1.02)	**83.66(0.72)**^a^
	KNN	78.32(0.99)	77.08(0.80)	**80.68(0.90)**^a^	70.37(1.17)	78.32(0.80)
	AdaBoost	77.39(2.07)	78.94(1.86)	70.87(1.32)	76.15(1.38)	**79.63(1.37)**^a^

^a^The best mean scores of percentage accuracy for each row is highlighted in bold font

Interestingly, during the utilization of classifiers, we had performed hyper parameter tuning for some possible classifiers estimated classification accuracy for each turning of the parameter. For example, for the KNN classifier, the number of neighbors (K) is tuned from 1 to 15 with step size 1, while for the C4.5 classifier, the confidence parameter (C) is tuned from 0.05 to 0.5 with step size 0.05, but for the Naive Bayes and Adaboost classifiers, there are no hyper parameters that need to be tuned. Finally, the best result among all sets of tuned parameter is considered for that specific classifier. For the first dataset (Exp), the SVM-RFE-CBR algorithm performs better than all other algorithms for all classifiers except KNN. For this dataset, the mRMR algorithm produces the best accuracy (=89.19%) for the KNN classifier. For the second dataset (ExpExon), the VWmRMR algorithm performs better than all other feature selection algorithms for all classifiers except the Naive Bayes classifier. For this dataset, the VWmRMR method achieves the best accuracy (=87.83%) for the KNN classifier. For the third dataset (hMethyl27), the VWmRMR algorithm performs better than all other algorithms except SVM-RFE-CBR for two classifiers, namely, Naive Bayes and AdaBoost. For this dataset, the SVM-RFE-CBR obtains the best accuracy (=84.41%) for the KNN classifier. For the fourth dataset (Gistic2), the mRMR algorithm yields better than all other algorithms for all classifiers other than KNN classifier. For this dataset, the mRMR algorithm obtains the best accuracy (=78.70%) for the AdaBoost classifier. For the last dataset (Pathway activity), the VWmRMR algorithm performs better than other two algorithms, INMIFS and SVM-RFE-CBR for all classifiers. For this dataset, the VWmRMR algorithm obtains the best accuracy (=83.66%) for the Naive Bayes classifier. As an overall, the VWMRmR method offers best accuracy in seven cases. On the other hand, the mRMR and SVM-RFE-CBR methods yield the best accuracy in six cases. Thus, the the VWMRmR method seems to be the best among all five feature selection algorithms in terms of providing the best accuracy in most number of times.

**Table 3:** Summary of the comparative performance of the proposed feature selection algorithm against other feature selection algorithms

Dataset	Criteria	mRMR	INMIFS	DFS	SVM-RFE-CBR
Exp	W-D-L (VWMRmR against other^a^)	2-0-2	1-1-2	4-0-0	1-0-3
ExpExon	W-D-L (VWMRmR against other^a^)	3-0-1	4-0-0	4-0-0	3-0-1
hMethyl27	W-D-L (VWMRmR against other^a^)	3-0-1	3-0-1	4-0-0	2-0-2
Gistic2	W-D-L (VWMRmR against other^a^)	0-0-4	1-0-3	3-0-1	0-0-4
Pathway activity data	W-D-L(VWMRmR against other^a^)	2-1-1	4-0-0	2-0-2	4-0-0

^a^Other is the feature selection method denoted by each specific column (e.g., mRMR in third column)

The comparative performances of the VWMRmR method against each of the remaining four feature selection algorithms is summarized in Table 3. For each data, the value of a cell under the column of a given feature selection algorithm specifies the number of times the VWMRmR method wins, draws and looses against the respective feature selection algorithm on that particular data. For the first dataset (Exp), the VWMRmR method wins in all four cases against the DFS method, whereas the VWMRmR method wins against the mRMR method the same number cases (=2) , whatever the mRMR method wins against the other. For this data, both the INMIFS and SVM-RFE-CBR methods wins against the VWMRmR method in more number of cases whatever the VWMRmR method wins against each of the them. For the second dataset ExpExon, the VWMRmR method wins in all four cases against two methods, viz. INMIFS and DFS methods. For this data, the VWMRmR method also wins against each of other two methods: mRMR and SVM-RFE-CBR, in three cases out of four cases. For the third data (hMethyl27), the VWMRmR method wins against the DFS method in all four cases, whereas it wins against each of two methods, namely, mRMR and INMIFS, in three cases. For this data, both the SVM-RFE-CBE and VWMRmR methods win against each other the same number of times (=2). For the fourth data (Gistic2), the VWMRmR against the DSF method in three cases. This is the only one data for which any other method (viz., mRMR and SVM-RFE-CBR) wins against the VWMRmR method in all four cases. Furthermore, for this data, the DFS method wins against the VWMRmR method in three cases. For the fifth data (Pathway activity data), the VWMRmR method wins against each of two methods, viz, INMIFS and SVM-RFE-CBR, in all four cases, whereas it wins against each of other two methods, viz., mRMR and DFS, in two cases. As an overall, the VWMRmR method performs better than other algorithms for most of the datasets.

#### **Comparison of different feature selection algorithms in terms of redundancy rate**

In this section, the performance of the aforesaid five feature selection methods is compared in terms of average redundancy rate. For this purpose, two different kinds of similarity measures are used. The first measure is the normalized mutual information and the second measure is the Pearson correlation coefficient. The comparative results of the average redundancy rate, in terms of normalized mutual information, of the top-ranked 50 features selected using different feature selection methods are shown in Table 4. In the table, for each data, the boldfaced entry under a given feature selection algorithm indicates that it offers the least redundancy rate among all feature selection algorithms on the respective data. The lower the value of redundancy rate, the better the selection of the feature selection algorithm is. As revealed from the table, the feature subset obtained by the VWMRmR approach is observed to be the least redundant in three cases. This shows that the VWMRmR algorithm is able to remove redundant features while selecting a small subset of relevant features.

**Table 4:** Average redundancy rate in terms of normalized mutual information (denoted as $RR_{avg}^{mi}$) of different subsets of features selected using various algorithms. Least value of $RR_{avg}^{mi}$ signifies better choice of the feature selection algorithm

Dataset	Feature selection algorithm				
	mRMR	INMIFS	DFS	SVM-RFE-CBR	VWMRmR
Exp	0.0978	0.1053	0.0988	0.1053	**0.0948**^a^
ExpExon	0.0972	0.0991	**0.0941**^a^	0.105	0.0961
hMethyl27	0.0984	0.1	0.1045	**0.0888**^a^	0.0894
Gistic2	0.3159	0.215	0.4258	0.3209	**0.1958**^a^
Pathway activity data	0.0659	0.0533	0.0778	0.1215	**0.0487**

^a^Bold font justifies least $RR_{avg}^{mi}$ among all algorithms (i.e., best performance) for each data (row)

Similarly, the comparative results of the average redundancy rates, in terms of the Pearson correlation coefficient, of the top-ranked 50 features obtained using the same five feature selection methods across all datasets are reported in Table 5. Similar to the above case, the lower the value of redundancy rate, the better the feature selection algorithm is. As revealed from Table 5, the VWMRmR method yields a small set of relevant features with the least redundancy for two datasets, viz., Gistic2 and Pathway activity data. On the other hand, the SVM-RFE-CBR method offers the least redundant feature sets for two datasets: Exp and hMethyl27, while the DFS method yields the least redundant feature set for ExpExon data. As an overall, the VWMRmR method is capable of removing redundant features to improve the learning performance.

**Table 5:** Average redundancy rate in terms of Pearson correlation coefficient (denoted as $RR_{avg}^{pc}$) of different subsets of features selected using various algorithms. Least value of $RR_{avg}^{pc}$ signifies better choice of the feature selection algorithm

Dataset	Feature selection algorithm				
	mRMR	INMIFS	DFS	SVM-RFE-CBR	VWMRmR
Exp	0.0281	0.0345	0.0145	**0.0124**^a^	0.0219
ExpExon	0.0258	0.0378	**0.0177**^a^	0.0545	0.025
hMethyl27	0.109	0.1235	0.1324	**0.0754**^a^	0.084
Gistic2	0.2953	0.1665	0.3244	0.2437	**0.1277**^a^
Pathway activity data	0.042	0.0291	0.0467	0.0684	**0.025**^a^

^a^Bold font justifies least $RR_{avg}^{pc}$ among all algorithms (i.e., best performance) for each data (row)

#### **Comparison of different feature selection algorithms in terms of representation entropy**

Table 6 shows the representation entropy of different feature subsets selected using the aforesaid five feature selection algorithms for various datasets. The value of each cell under i-th row and j-th column represents the representation entropy of the top-ranked 50 features obtained by applying the j-th feature selection algorithm to the i-th dataset. In the table, the boldfaced value for each entry indicates that the corresponding feature selection algorithm is the best among all algorithms in terms of providing the maximum representation entropy. The higher the value of representation entropy, the better the feature selection algorithm is. As revealed from Table 6, the VWMRmR algorithm obtains the best representation entropy for three datasets, viz., Exp, Gistic2 and Pathway activity data. On the other hand, the DFS and SVM-RFE-CBR algorithms give the best representation entropy for the remaining two datasets, viz., ExpExon and hMethyl27, respectively. For both these two data, the VWMRmR method offers the second maximum value. As an overall, the VWMRmR method seems to be the best in terms of obtaining the best representation entropy in most cases.

**Table 6:** Representation Entropy (*RE*) of feature subsets obtained using different supervised feature selection algorithms. The higher value of representation entropy is the better choice of the feature selection algorithm

Dataset	Feature selection algorithm				
	mRMR	INMIFS	DFS	SVM-RFE-CBR	VWMRmR
Exp	4.4272	4.3292	4.4012	4.3777	**4.4731**^a^
ExpExon	4.4364	4.3815	**4.4975**^a^	4.4418	4.457
hMethyl27	4.3622	4.3389	4.3951	**4.4596**^a^	4.4462
Gistic2	2.275	2.9071	1.5355	1.9769	**3.2265**^a^
Pathway activity data	4.7957	4.8386	4.3463	4.0656	**4.8581**^a^

^a^Bold font justifies the highest *RE* among all algorithms (i.e., best performance) for each data

#### **Intersection of features obtained by different feature selection algorithms**

**Fig. 2:**
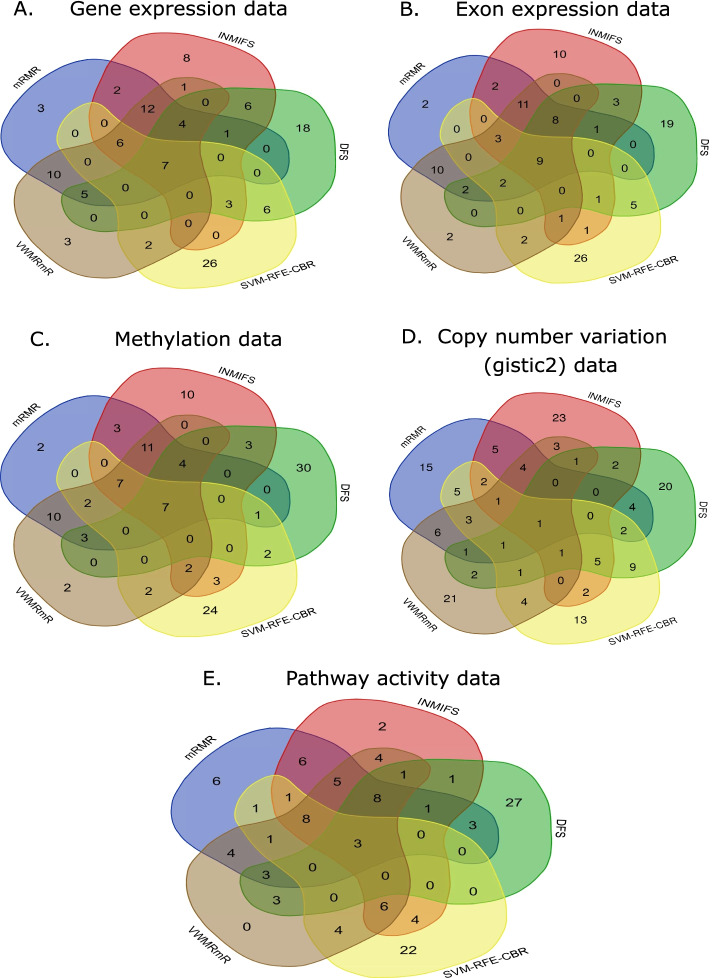
Venn diagrams showing the intersection of top 50 extracted statistically significant features (genes) among five feature selection algorithms: **A** expression data, **B** exon expression data, **C** methylation data, **D** copy number variation (Gistic2) data, and **E** pathway activity data

In addition, we carried out the intersection of top 50 extracted statistically significant features (genes) among five feature selection algorithms and also represented Venn diagrams for expression data (Fig. 2A), exon expression data (Fig. 2B), methylation data (Fig. 2C), copy number (Gistic2) data (Fig. 2D), and pathway activity data (Fig. 2E).

For gene expression data, we obtained 7 common genes among the five feature selection methods (mRMR, INMIFS, DFS, SVM-RFE-CBR and VWMRmR). Those were *ZMIZ1, ENG, FGFR1, PAWR, KRT17, MPO* and *LAT2*. For exon expression data, we obtained 9 common genes among those five feature selection methods (mRMR, INMIFS, DFS, SVM-RFE-CBR and VWMRmR). Those were *ZMIZ1, ENG, ALOX15, RTN4R, FGFR1, KRT17, PTPRM, MPO* and *LAT2*. In the analysis of methylation data, 7 overlapped genes (namely, *PRF1, FCGR2A, CD3D, MPO, ANGPT1, PREX1* and *KRT17*) were identified for those feature selection methods. For copy number (Gistic2) dataset, we identified only one gene (*PIK3CG*), while 3 genes had been found for those feature selection methods for pathway activity data (namely, *BMP4, STXBP1* and *LEP*).

## **Conclusions**

In this paper, we presented a comparative study of five widely used state-of-the art feature selection methods such as mRMR, INMIFS, DFS, SVM-RFE-CBR, and VWMRmR for multi-omics datasets. The usefulness of different feature subsets selected using the aforesaid five feature selection algorithms was assessed using three evaluation criteria: classification accuracy, representation entropy and redundancy. For the comparison purpose, four different classification methods such as C4.5, Naive Bayes, KNN and AdaBoost were used to measure the worth of each selected feature subset. Overall, the VWMRmR method offers the best performance for all three evaluation metrics for majority of datasets. The VWMRmR algorithm obtains the best *Acc* for most of the cases across all datasets except the Exp and Gistic2 data. It also obtains the best *RE* for three datasets (Exp, ExpExon and Paradigm IPLs); while the DFS and SVM-RFE-CBR algorithms produce the best *RE* for the remaining two datasets - hMethyl27 and Gistic2, respectively. Additionally, we carried out intersection of top 50 statistically significant features obtained by those five feature selection methods for each omic data and overlapped feature set is defined as signature genes using supervised learning. We obtained a 7-gene signature (*ZMIZ1, ENG, FGFR1, PAWR, KRT17, MPO* and *LAT2*) for gene expression, a 9-gene signature for exon expression data, a 7-gene signature for methylation data, a single-gene signature (*PIK3CG*) for copy number data and a 3-gene signature for pathway activity data. In future, we plan to develop sophisticated feature selection algorithms to speed up the process of feature selection for such kinds of datasets with more than ten thousand samples. We have also planned to incorporate higher order dependencies among features.

## Data Availability

The following publicly available databases have been used in this study namely xenabrowser database (https://xenabrowser.net/datapages/?cohort=TCGA)

## Abbreviations

INMIFS: Improved normalized mutual information feature selection
MIFS: Mutual information-based feature selection
mRMR: Minimum redundancy maximum relevance
NMIFS: Normalized mutual information feature selection
VWMRmR: Variable weighted maximal relevance minimal redundancy
